# Crafting representations of rare disease: collage as qualitative inquiry

**DOI:** 10.1080/17533015.2023.2254328

**Published:** 2023-09-11

**Authors:** Richard Gorman, Bobbie Farsides, Maria Bonner

**Affiliations:** a Department of Clinical and Experimental Medicine, Brighton and Sussex Medical School, BSMS Teaching Building, University of Sussex, Brighton, UK; b Independent Artist, UK

**Keywords:** Collage, arts-based research methods, rare disease, creative methods

## Abstract

**Background:**

Collage is a modality of expression which involves repurposing and juxtaposing fragments. Our aim was to explore both how and what collage, as an arts-based research method, might contribute to enlivening understandings of the experiences of families affected by rare conditions.

**Methods:**

During 10 weeks of collaging workshops participants created artistic representations of their experiences. The methodology produced a convivial atmosphere where participants talked openly about everyday challenges.

**Results:**

The collages and conversations produced offer a means through which to consider the complex and multiple positions which families affected by rare disease interpolate. Particularly, the collages prompt cross-cutting thematic reflections on motherhood and care, the challenges of being heard, and balancing family life alongside medicalisation.

**Conclusions:**

The opportunity to convey topics and feelings through a medium which was both tentatively open yet conceptually complex allowed the broaching of sensitive and elusive themes in a safe, expressive, and non-threatening manner.

## Background

Research on the interrelationships between arts and health is an established and dynamic field of interest, with a growing body of evidence showcasing the value the creative arts can bring to healthcare (Fraser & Al Sayah, 2011). Artistic approaches have been mobilised as part of health promotion, empowerment, and behaviour change campaigns, and are increasingly recognised as playing an important role in the training of healthcare professionals (Clift & Camic, 2016). The arts are also becoming a valuable method within health research itself, particularly as an innovative means of both knowledge production and knowledge dissemination. Arts-based approaches can enhance health research by illuminating and elucidating “human dimensions of health and illness in ways that augment our understanding of health and social care” (Boydell et al., 2012, p. 2).

Drawing on an arts-based health research framework, we have been working with families affected by rare genetic conditions to explore ways of capturing and sharing important stories and messages about their lives and to situate their narratives and experiences against and amongst “the myriad stories already told about their lives by ‘experts’” (Liddiard, 2018, p. 11). In doing so we hope to prompt healthcare professionals to be more “prepared” for encounters in the clinic (Farsides & Lucassen, 2023). Having already explored textual and filmic forms of storytelling (Gorman et al., 2022; Gorman and Farsides, 2022), and encouraged by our participants, we were intrigued by the possibilities offered by visual methodologies – particularly collage – which offers valuable potential in creating space for different knowledge and modes of representation. As Rose (1997, pp. 306–307) argues, “the sort of knowledge made depends on who its makers are”. Our aim has been to explore both how and what collage, as an arts-based research method, might contribute to enlivening understandings of the experiences of families affected by rare conditions. As such, we were interested in the methodologies of practice, the experiences of making, and the representative artefacts produced.

### Collage as art

Collage is the practice of using fragments of found images (or various other materials) and gluing them to a flat surface to portray phenomena. The phrase “collage” comes from the French verb *coller*, meaning to stick. Pablo Picasso and George Braque are frequently credited with introducing collage as a more formal mode of artistic expression through Cubist experiments in dismantling traditional realistic representations (Raaberg, 1998). However, paper collage as a folk practice has a lengthy and rich history and Schapiro and Meyer (1977) have challenged the conventional genealogy locating collage’s origins with Picasso and Braque. They note that many artists made collages before the word “collage” was “invented in the twentieth century to describe an activity with an ancient history” – an activity often undertaken by non-Western artists, women artists, or anonymous folk artists who have been excluded in art history accounts.

Collage became a major modality of expression and experimentation across the arts of the 20th century. It was particularly seized upon by the Dadaists as a strategy for cultural critique; “a means of undermining conventional associations and shocking the viewer or reader into a perception of a new reality – social, political and psychological, as well as aesthetic” (Raaberg, 1998, p. 155). Collage as an artform and practice allowed female artists such as Marianne Brandt and Hannah Hoch to “perform an original female point of view” and “insert their point of view in the social imaginary and propose their own self-representation” and in doing so overturn a traditional male-oriented perspective (Toschi, 2013, p. 326). Excluded from the metal workshops they sought to create their own influence upon the Bauhaus movement.

The conscious use of collage as an art form was part of modernist artists’ rejection of the use of purely orthodox materials, techniques, and processes and the embracing of the unorthodox, challenging the art canon. Feminist art practice has always underpinned avant-garde practices, particularly in postmodernism, but collage predates this. As Lippard (1995, p. 168) describes,

Collage is born of interruption and the healing instinct to use political consciousness as a glue with which to get the pieces into some sort of new order (though not necessarily as new whole, since there is no single way out, nobody who’s really ‘got it all together’; feminist art is still an art of separations)

The associations between collage and women’s art activities can be seen in the concept of “femmage”, or, feminist collage, a concept coined by Schapiro and Meyer (1977). As Raaberg (1998, p. 157) explains, femmage is:

“A strategy in the traditional women’s art activities of handiwork and crafts, which in the past had provided a felicitous method for women with limited access to the means and materials of the ‘fine arts’ to create beautiful and useful art […] marked by collage techniques of piecing together fragments […] saved and recycled materials, combining available materials, photographs, printed matter, images, and texts into abstract and organic forms; they produced, for an audience of intimates, works that were both functional and aesthetic, that had reference to both private and public events, and that were often subversive.”

By centring traditional women’s techniques – sewing, embroidery, quilting, appliqué, and photomontage, femmage helps redefine the way we think about art and representation through its use of diverse materials and perspectives on womanhood (Balducci, 2011). In our necessarily brief introduction to collage as art here, we would stress the medium’s use by “artists who are culturally marginal” (Raaberg, 1998, p. 154) as a strategy for manifesting art with political entanglements. This was how collage was introduced and contextualised to our participants and it was these traditions which particularly resonated with them as inspiration for what would become their own work.

### Collage as research

The last 20 years have seen a rich and vibrant interdisciplinary literature develop relating to, and reflecting upon, the use of collage as a method. Collage now has a strong tradition as a mode of qualitative inquiry, developed to increase the possibilities for including different voices in research, and enabling space for multiple representations, realities, and understandings (Butler-Kisber, 2008). Yet, there remains something of a tendency to view collage as a practice or process to be used to help researchers themselves grapple with, or present, ideas (Butler-Kisber et al., 2003). Collage in this way becomes a mode of *analysis*, rather than a *method* to procure “data” itself (Butler-Kisber et al., 2003); an activity that is retained as something artist-researchers do to other participant-generated qualitative data. We wanted to move beyond this model to consider collage as a method of inquiry utilised *with* participants (Mackworth-Young et al., 2020; Vacchelli, 2018): collage as a participatory method. Butler-Kisber et al. (2003, p. 139) call for more research that uses “collage in a collaborative context”, working with participants to garner insights.

Collage, when mobilised as a form of qualitative inquiry, allows for multiple ways of looking at phenomena (Butler-Kisber et al., 2003), providing a visual representation and synthesis of feeling and thought, allowing for illuminating relations of juxtaposition and difference. Whilst retaining a level of ambiguity it provides “a way of expressing the said and the unsaid, and allows for multiple avenues of interpretation and greater accessibility” (Butler-Kisber, 2008, p. 268). Pictorial presentations can expose factors which remain elusive to discursive methods (Williams, 2000). Similarly, the possibility for the incorporation of “found objects” (tickets, receipts, packaging) enables an artist to “tell stories through a direct visual connection to the real world of lived experience” (Klorer, 2014, p. 147). Importantly, collage allows a level of agential self-representation, which can be particularly valuable when narrating experiences where an ability to express agency has been lacking. There is a level of safety in this ambiguity, a tentative way to explore complex ideas which may be emotive, sensitive, and resistant to the definitude of speech or written forms. Through choosing and organising imagery, participants retain control of the constitutive elements of their stories, the pace of disclosure, and how they choose to present or compartmentalise selves (Debbink et al., 2016). Cartwright, writing in Hasbun et al., (2022), has reflected on collage as a trauma-sensitive practice, one which disrupts expectations of linear narratives in favour of openness.

By constructing narrative through the synthesis of fragments collage “arrives at meaning in a very different way – accidentally, capriciously, provocatively, tangentially” (Davis, 2008, p. 250) combining multiple meanings that are individual, internal, and personal alongside those that are sociocultural, external, and contextual (Bell, 2013). The issue of meaning is important, given the need to separate, or at least, understand, both the meaning intended by the artist and the meaning bestowed by the viewer. Collage has a level of potential in producing multivocal and nonlinear representations (Butler-Kisber, 2008), offering a way to disrupt notions of a “purity” of representations, accounting for the intersection of multiple discourses (Kangas et al., 2019). Collage as a method of inquiry is a “means to intertwine layers of knowledge” (Chilton & Scotti, 2014, p. 166) bringing together and making visible disparate ways of knowing and different aspects and layers of identities. For Bell (2013, p. 233), collage mobilises an intertextual approach, that “creates an intriguing interstice between the signifiers and the possible realities they portray”. Meaning is created not (just) from the images themselves, but through the way they (are seen to) relate to one another (Butler-Kisber, 2008), what Garoian (2004, p. 25) describes as the “dialectical interplay of conflicting signifiers” leading to “creative cognition under the circumstances of collage”. Vacchelli (2018) argues collage has a methodological utility as it values pre-conscious intuitions as a means to generate data, allowing an engagement with, and emphasising of, how non-representational processes might shape how life unfolds (Gorman & Andrews, 2022).

Alongside what is *produced* as collage, the *process* of collage-crafting is important and revelatory. It is through the very process of *doing* and *making* of collages that thoughts, ideas, and knowledges surface, emerge, and develop (Bell, 2013). Collage as method involves experimentation and discovery. It is a process of “opening up the possibility for the emergence of tacitly or intuitively known content and the appearance of unexpected new associations” (Davis & Butler-Kisber, 1999, p. 4). The “hands-on” experience of physically cutting and gluing lends a level of embodiment to the method and production of knowledge (Chilton & Scotti, 2014; Vacchelli, 2018). Particularly, the reconfiguring of “found” elements allows the challenging of inflexible, prescriptive, and hegemonic representations and definitions (Davis, 2008). In this way collage creates a way to explore and reflect upon “the effects of our immersive multimedia environment” (Davis, 2008, p. 263) on identity and subject-positions and allows participants to disrupt normative ways of depicting and being depicted (Plakoyiannaki & Stavraki, 2018). Collage repoliticises images, inviting viewers to pay critical attention to how normative images circulate in the everyday whilst also noticing what is absent (Särmä, 2018).

Practically, collage has a level of accessibility that many arts-based methods lack (Butler-Kisber, 2008). As Williams (2000, p. 274) argues, “the value of collage over many other forms rests with the notion that it is a non-threatening medium where an individual does not need to feel ‘artistic’ in producing their piece of work”. Of course, the accessibility and intuitiveness of the practice also create challenges, as Bell (2013, p. 237) notes, “it is far easier to create a pleasing ‘picture’ rather than an unsettling visual response full of dissonance” and the making of collage requires thoughtful consideration. Indeed, Burge et al., (2016, p. 734) describe how arts-based research methods can alienate, baffle, or discomfort participants, and how people “may feel that their status as adults and as experts is at risk” when unfamiliar and unconventional modalities of expression are mobilised.

Finally, collage as a research method allows for discourse and dissemination (Bell, 2013). The evocative and visually poignant nature of collage can trigger new affective responses (Davis & Butler-Kisber, 1999) and lead both artist and viewer (or, research audience) to be provoked “into new ways of knowing” (Butler-Kisber, 2008, p. 268). Collage “encourages new ways of looking” (Bell, 2013, p. 224) and allows for a different kind of dissemination, one that is more participatory. As Gerstenblatt (2013, p. 306) notes, participatory-collage research moves “beyond the traditional research outcome of publication in a journal participants may never read to sharing the results of the inquiry and feeling a part of the process from beginning to end”. Creating such an ethos of active-engagement, inclusion, and partnership in research can change the very relations and conversations that ensue, allowing for different forms of dialogue and more diverse understandings. The possibility for such different forms of dialogue resonated well with our aims to explore experiences that are sensitive, emotional, and resistant to representation, and convey these to audiences of healthcare professionals, that they might garner additional affective knowledges applicable to their clinical practice.

## Research approach and methodology

As part of a larger research programme (granted ethical approval by the Brighton and Sussex Medical School Research Governance and Ethics Committee), we worked with regional rare disease networks and support groups to recruit participants with lived experience of rare genetic disease in their families. Participants were parents of children with different conditions resulting in complex physical and cognitive impacts, facing the challenges of navigating often oblique pathways to access clinical and social care support. This group has shaped our research throughout, guiding our development of questions, methods, and dissemination. Out of this group (comprising 12 regular attendees, both mothers and fathers), seven participants (all female, all mothers) expressed an interest in utilising collage as a way to explore different ways of understanding, (re)presenting, and evoking their experiences. We had hoped to encourage more diverse participation in terms of gender, but despite our best efforts, this was not to be. However, we don’t feel we can draw any conclusions about collage necessarily being more appealing to mothers, though we did note a real enthusiasm for understanding the history of the approach as a feminist art practice with links to traditional women’s art. Other scholars exploring the experiences of parents of children with rare diseases have similarly noted a prevalence towards participation and engagement from women, and have called for more work that interrogates the intersection of gender and caregiving in the worlds of rare disease (Baumbusch et al., 2018; Gorman et al., 2022). Our discussions on motherhood which follow later, begin to explore this.

We approached a local artist with expertise in collage art. Working together, we designed and recorded two instructional videos introducing participants to the medium, historically, artistically, politically, and practically. We contextualised the history of collage, noting its use as a means of political commentary, often by underrepresented groups. This contextualisation proved to be hugely important. It both legitimised the method, and gave our participants a sense that they were connecting to a larger tradition and culture – particularly to collage as a traditional women’s art form and a response to exclusion. These resources also covered issues of technique, and the different effects and resonances that could be created from tearing, cutting, and assembling source images.

After they had watched the introductory videos we arranged weekly workshops with our participants online via Zoom to work on collages. Online mediums are now well established as enabling positive researcher-participant relationships and allowing for the collection of rich and rigorous data. Working online allowed us to engage with a more geographically dispersed group of participants with significant caring responsibilities, and engage with those affected by particular vulnerabilities in pandemic times. Thus, whilst online research can be exclusionary, for us, it served as a technology of inclusion. Whilst there are concerns about the intrusiveness of video-technologies, the option to “blur” or have artificial backgrounds also allows people to feel more comfortable in a familiar home environment, and created an ethos of inclusivity for those who sometimes needed to disappear off screen to attend to family responsibilities – people who would normally not be able to participate in in-person events.

Online research can sometimes leave participants feeling disconnected from the researchers though, so ahead of meetings, we sent participants a resource pack containing a cutting mat, a craft knife, glue, scissors, tweezers, paper, card, and a sketch pad. We encouraged participants to stockpile magazines, junk mail, and other image-rich media, along with “everyday paperwork”, whether travel tickets, receipts, or hospital detritus. We also offered participants a budget to acquire magazines.

With participants’ consent, we recorded the workshops, allowing us to reflect on the kinds of conversations collaging together produced. The sessions proved popular, and ended up running for 10 weeks – much longer than we had planned, but participants reported valuing the opportunity to come together both to work on their pieces but also to talk around and about them. Participants were reminded that it was for them to decide on a case by case basis what they wished to share (we know that some work was produced which wasn’t shared with the group or the researchers), the importance of respect for different people’s stories and creative outputs, and the need for sensitivity and discretion. Throughout the project we have remained mindful of the family dimension to the stories told and have discussed directly and indirectly with participants particular choices and decisions relating to sharing images or words relating to identifiable others.

Participants would join and parse through piles of magazines and other media, tearing, cutting, and collecting fragments of images and words that attracted them. The informal nature of the activity, as well as its relative ease, produced a convivial atmosphere where participants chatted openly. Meeting regularly allowed a sense of community to emerge, and gave us an ethnographic insight into participants’ lives. People produced collages at different paces, but were always keen to share their creations with others. An instant messaging group was set up amongst participants to allow for conversations to continue between sessions, and to facilitate sharing. We also set up a digital collaborative space where people could upload their finished artworks.

Given that we were exploring topics relating to often life-limiting conditions, along with signposting support organisations, we also engaged a colleague with professional training in palliative care settings and extensive pastoral experience who could be called upon to support participants and researchers should the experience become challenging. There was also extensive mutual support between participants, who found a sense of solidarity in sharing their experiences and their work. Participants also engaged in reflexive self-censorship and curatorial care-work themselves. For example, the artist behind Figure 3 decided not to share this collage with the wider participant group whilst the sessions were on-going, as they were aware it could be upsetting for some. However, they were also keen for the artwork, and its messages, to be shared as part of our research in this more public space. It speaks to the ethos of the group culture and community which had emerged through communal collaging.

The collages were not just means by which to initiate conversations, but rather “conveyors of meaning in their own right” (Roberts & Woods, 2018, p. 631). Though we were impressed by the conversations and subsequent insight that collage as a method produced, we were also committed to using the produced collages as more-than just secondary illustrations. We engaged with ongoing debates on how to appropriately analyse the artefacts produced through arts-based-research-methods. Chilton and Scotti (2014) advocate drawing on thematic analysis – a qualitative research method aimed at identifying, analysing and reporting patterns of meaning (Braun & Clarke, 2006) – to understand collage. Rose (2001, p. 32) argues a critical approach to visual images is needed, “one that thinks about the agency of the image, considers the social practices and effects of its viewing, and reflects on the specificity of that viewing by various audiences”. Building on Rose, and with collage-methods specifically in mind, Butler-Kisber (2018, pp. 121–122) suggests asking:

What overall feeling does the collage express? What do the compositional aspects of the collage suggest (line, colours, spaces, directionality, foreground/background)? What metaphorical meaning(s) emerge(s) in the collage? How do the images used and the perspective of the collagist contribute to the message? How does the perspective of the viewer contribute to the response to the collage? In sum, what overall message does the collage express?

Here we offer a thematic analysis and interpretation of the prominent themes identifiable across the collages produced. With participants’ consent we have chosen to display three collages. Throughout our project, participants have retained the right to decide how their work is shared and some opted not to share their creations beyond the group. Importantly, the collages cannot be seen as separate or disentangled from the participants’ lived experience as carers for their children with rare diseases, so we also draw on some segments of conversation with our participants. Transcripts from the group discussions were similarly thematically analysed. As Culshaw (2019, p. 272) argues, it is important to think about the intermingling of visual and verbal data, with the dual processes of creating and then later explaining collages helping participants to “better express their stories” and “arrive at understandings they may otherwise have missed”. Whilst acknowledging the intentionality of an image’s maker is a vital part of visual analysis (Rose, 2001), what emerged from the verbal data was a sense that what the various creators had intended to make was a “provocation” for healthcare professionals and a point of connection for other parents, rather than definitive accounts or statements. Thus whilst some, such as the artist behind Figure 3, did allude to their intent (*“It’s supposed to kind of chart – in a very simplified way – my feelings of guilt from my son’s birth (well, actually pre-birth) to death”*), others were keen to leave matters of definitive interpretation open, and thus though we offer our analysis and interpretation – supported by conversations with participants – we also offer the collages to readers to make of them what they will.

## Results

The collages offer a means through which to consider the complex and multiple positions which families affected by rare disease interpolate. Particularly, when read together, the collages prompt cross-cutting thematic reflections on motherhood and care, the challenges of having a voice and being heard, and balancing family life and medicalisation.

## Theme 1: ‘motherhood’

Motherhood can be seen as an important theme cutting across all of the collages. It was a regular topic amidst the background chatter of the collaging workshops as participants reflected on the struggles they had encountered in both maintaining and challenging identities as mothers. The recorded sessions also reveal interruptions due to demands of motherhood, ranging for caring for a sick child to receiving calls from medical services. Literature has indicated that mothers of children with rare diseases often “serve as primary caregivers and perform intensive and time-consuming physician-prescribed treatments and home care tasks” (Chu et al., 2022, p. 10), yet there is little scholarship that explores how this remaps emotional landscapes and identities for women.

**Figure 1.**
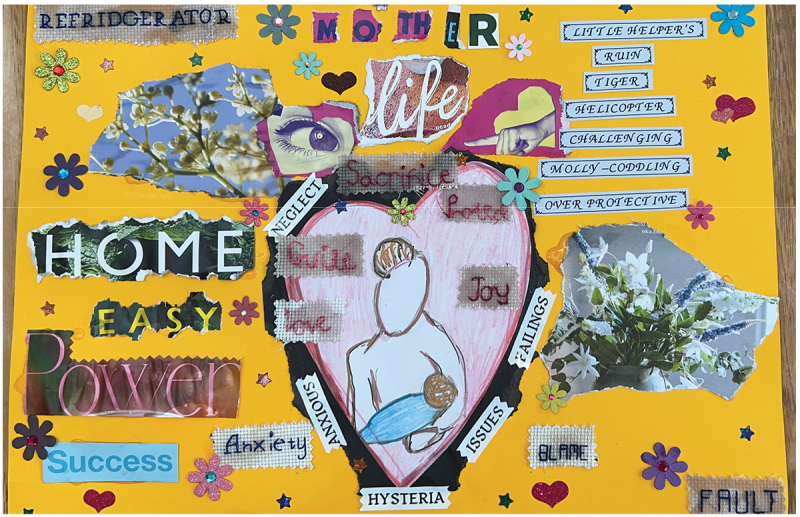
“Mother life”, a collage produced by a participant.

Figure 1, “Mother Life”, in particular takes up the challenge of presenting an account of the intersections between motherhood and the everyday experience of life with rare disease. By playfully and critically incorporating the vocabularies often applied to mothers, the collage highlights the gendered pejoratives that can creep into, and shape, the caregiving experience: molly-coddling, tiger-mother, over protective. These labels – literally printed with a label-maker – contrast with the participant’s inner feelings which are instead picked out in needlework. Talking about her inspiration for putting this artistic commentary together, our participant reflected on her struggles to be taken seriously as simultaneously both a mother and a caregiver in a healthcare context:

I asked for her notes, but of course, even by doing that, it puts you up there as … so, we’ve got all the notes, and it says “Mother struggles with the idea of acceptance around her child being undiagnosed”, and “Mother was emotional when spoken to”, and phrases like “Mother is on support group”. Somehow you don’t have any validity … I’ve had to ask [partner] to come as well, because at least then, it’s not just “the hysterical mother”.

Also exploring the intersections between motherhood and rare disease, “Chart” (Figure 3) involves the conscious choice to incorporate personal and family photos as a way to centre the experiences and emotions encountered as a mother, juxtaposed and set free from the eclipsing nature of experiences which can become codified as solely medical. As the participant later explained:

It’s supposed to kind of chart – in a very simplified way – my feelings of guilt from my son’s birth (well, actually pre-birth) to death. Which is not to say there weren’t a lot of other feelings along the way, but I think guilt is a common one among mothers and particularly mothers of disabled children, and I wanted to acknowledge that.

**Figure 2.**
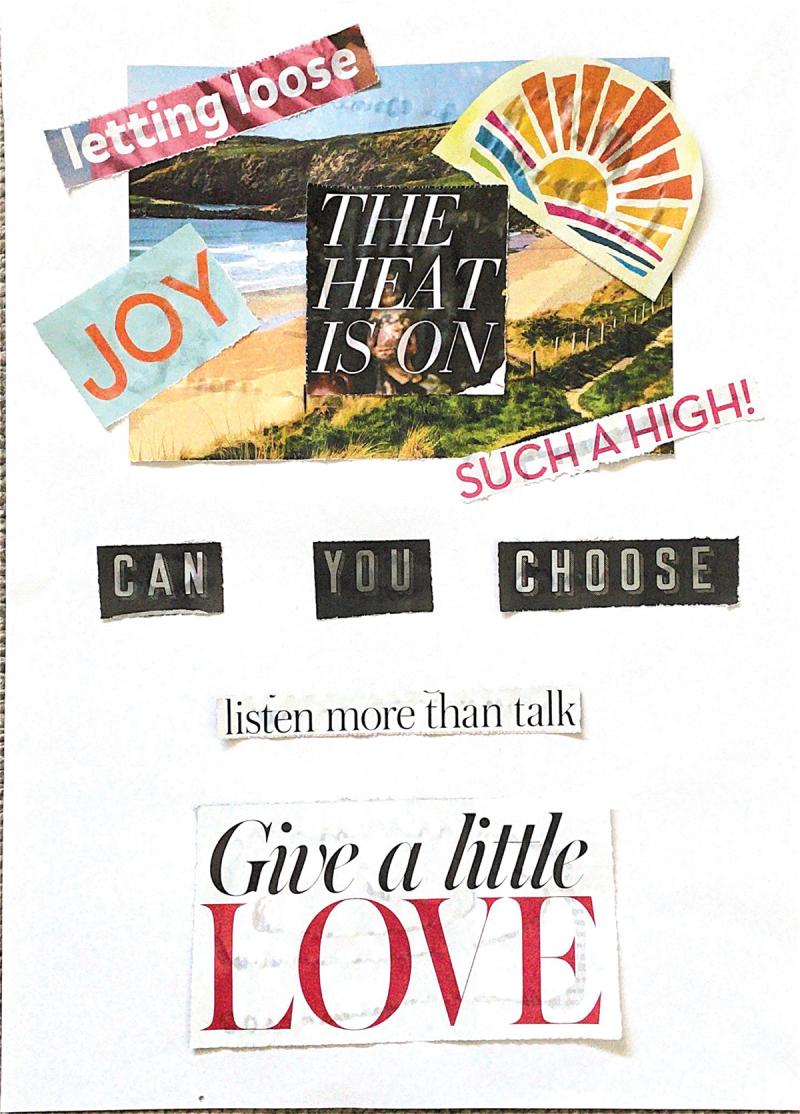
“Listen more”, a collage produced by a participant.

Running thematically across these collages is the sense that mothers often feel unheard when it comes to advocating for their children. The artist of “Mother Life”, quoted above, felt obliged to involve her partner, a man, in order to be properly heard thus revealing how gender cuts across experiences of care to render certain voices and experiences invalid and privilege others. Similarly, “Listen More” (Figure 2) the fonts and colours, immediately recognisable to anyone familiar with “women’s magazines” and the use of a sloganizing phrase “listen more than talk” becomes a plea to create more inclusive and accessible spaces where mothers’ expertise can be valued and shared. This shows how discussions of motherhood elided into the second theme running across these collages, “being heard”.

## Theme 2: ‘being heard’

There is a growing acknowledgement in the literature of rare disease that “parental narratives have been unvoiced, misunderstood and incoherent to health-care providers (HcPs) and the community at large, often leading to the silencing of these multivocal experiences” (Currie & Szabo, 2019, pp. 1251–1252). This emerged as a central overarching theme in the collages that our participants produced.

As mentioned above, the theme of “being heard” can be seen strongly in the collage “Listen More” (Figure 2). When shared amongst the group, the collage provoked a wide-ranging discussion about the need to disrupt clinicians’ cursory rhetoric and create space for caregivers’ voices and expertise. As one participant, reflecting on the collage, explained:

I think, sometimes, if you, as a patient, just give them permission to not know, and say look, it’s much more important that I feel heard.

**Figure 3.**
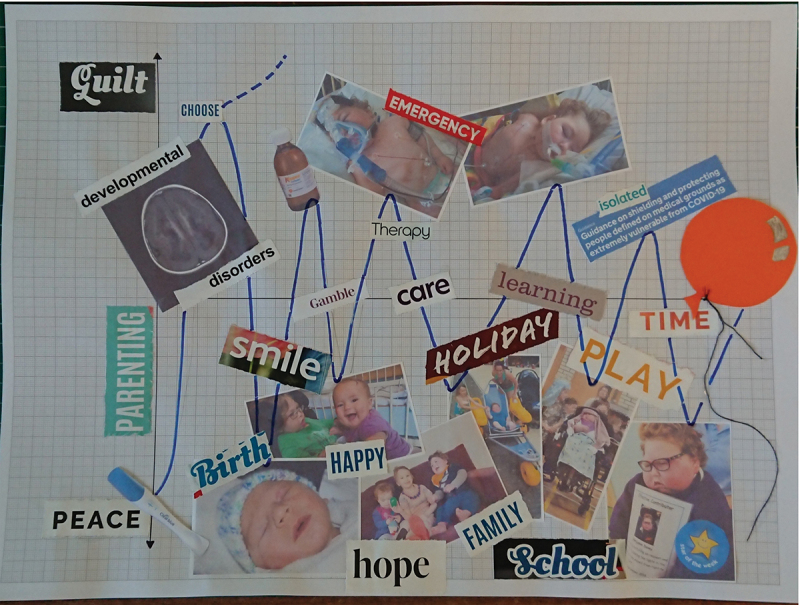
“Chart”, a collage produced by a participant.

When dealing with rare disease, health care professionals may feel uncomfortable in embracing uncertainty, yet, often, families are looking for empathy, rather than certainty. We can see this thematically expressed in “Chart” (Figure 3) which segments and juxtaposes lived experience and medicalised encounters in different quadrants of a graph. Clinical encounters can frequently leave little room for appreciating lived experience and the types of emotions and aspects of life that “Chart” draws attention too: hope, family, play, school, holidays. The theme of “being heard” we draw attention to here is not simply about listening to families, but rather finding ways to pay attention to the whole person at the centre of care, not just as clinical case to be solved.

When families, caregivers, and, to return to the prior theme, mothers, do not feel heard, this can result in distress (Chu et al., 2022) and feelings of guilt and anxiety can emerge, as can be seen with the phrases orbiting the collage “Mother Life” (Figure 1). This collage identifies how being unheard can devalue the care-work that families do, presenting and misrepresenting this care-labour as “hysteria” and “overprotective”. Being heard and overcoming epistemic injustice has important connotations for improving the quality of life for caregivers (Chu et al., 2022; Currie & Szabo, 2019). Whilst sitting and sifting through magazines for source material online, participants regularly chatted about being asked the same questions over and over again in healthcare settings – often in quite (re)traumatising ways – and feeling as though they had not been listened to:

You get asked those questions all the time, whenever you go to a new clinician, they always go through the whole history, pregnancy, and birth, they ask: did you drink? are you related to one another? Ask those questions one time and then put it at the front of the notes!

## Theme 3: balancing family life and medicalisation

All of the collages, at their heart, hold together a tension around balancing family life and medicalisation. As Belzer et al. (2022, p. 2) describe, many parents of children affected by rare disease report “facing a veritable medicalization of their homes and family lives”.

Interrogating this theme, “Chart” (Figure 3) utilises personal and family photos, juxtaposed against bold headlines, and organised via the scientific schema of a graph. The grid paper as background makes this messaging possible, whilst also acting as a metaphor for the constraints imposed on family life by current scientific and medical regimes of knowledge. The line graph, reminiscent of electrocardiographic readouts, shows our participant’s fluctuating experiences between feelings of peace and guilt. For us, as researchers interested in improving people’s experiences of accessing healthcare, it is telling that many of the more medicalised images are the ones assigned to the “guilt” quadrants, and we might wish to ask how healthcare professionals could be better equipped to support families in clinical situations where they are faced with nuanced ethical and emotive challenges for which their training will have given little preparation.

Particularly though, “Chart” (Figure 3) demonstrates the power of collage to enable people to tell stories which bring different narratives into conversation, setting the joys, vitality, and “normality” of family-life within medicalised framings. Unpacking this tension is also an important theme in “Listen More” (Figure 2) where “love” emerges as the largest text and concept that outshines any need to “choose”. The very concept of “choice” is one that looms large when it comes to disability, genetics, and ethics. The latter three topics may be visually absent from the artistic assemblage, but are intimately entangled through the positionality of our participants, the wider context of our research, and the conversations which accompanied the creation of these collages, as evidenced below:

When our first child was born, we didn’t have a diagnosis. They were like, we can talk to you about the chances of what would happen if you had another child … they’re obsessed with that … the whole ethos is that, it is a bad thing, but we love our daughter for who she is and we don’t want her designed out of existence, so, when you’re saying “oh the risk of this happening again”, you’re being very negative about a member of our family whom we love the way she is.

When read through the lens of caregiving and the complex nature of life with rare disease, the question of “can you choose?” posed by the collage “Listen More” (Figure 2) becomes particularly poignant, perhaps signifying the challenges a social model of disability creates for families in being able to access the spaces of relaxation at the heart of the collage. Belzer et al., (2022, p. 2) report that families of children with rare diseases often express feelings of social isolation and “not being understood by their peers with more typically developing children”. Similar themes can also be read from “Mother Life” (Figure 1) where phrases clipped from glossy magazines “easy”, “power”, and “success” juxtapose with more negative emotions and imagery of care-giving. Chu et al., (2022, p. 2) note a trend in families affected by rare disease where “mothers often left or changed their jobs to focus on caring for their child’s everyday needs”. Gender-based expectations overlay all of these other aspects and intersections between family life and medicalisation.

Through connecting and sharing in the political traditions of collage as a strategy for women to insert their point of view in the social imaginary, the collages have revealed important cross-cutting thematic reflections on motherhood and care, the challenges of having a voice and being heard, and balancing family life and medicalisation.

## Conclusion

Our goal throughout this process has been to empower our participants to share rich, vivid, and affective creative artefacts that might challenge, reveal, and provoke understandings of the experiences of families affected by rare conditions. Currie and Szabo (2019, p. 1257) call for more interpretive studies that “give voice to the parent perspective to improve quality of life, care practices, and public policies for families living with a rare disease”. The collages produced demonstrate the possibilities of collage-art as a way of empowering people to tell stories which are not always straightforward or amenable to easy phrasing or distribution. Collage serves as a platform that can facilitate people’s voices being heard and listened to. Utilising collage as a method of qualitative inquiry has been revealing in exploring how participants view their own experiences, freed from representational forms focused on linearity and language. The opportunity to convey topics and feelings through a medium which was both tentatively open yet conceptually complex, allowed the broaching of sensitive and elusive themes in a safe, expressive, and non-threatening manner. The contextual introduction to collage as a strategy with feminist traditions (Raaberg, 1998) was particularly important, providing participants a place in a recognisable oeuvre and a sense of empowerment in self-representation through art. We would encourage others intrigued by this method to account for the traditions at play here.

Much of the literature discussing collage-based research methodologies describes in-person events, with people bodily co-present in their cutting and sticking. The COVID-19 pandemic and the particular circumstance of our participants excluded the possibility of this for us, yet our remotely facilitated approach did not reduce the abilities of a collage methodology to secure high levels of engagement alongside interesting and rich outputs.

In our concluding remarks, we also want to reflect on the decision to publish Figure 3, which utilises a participant’s personal and family photos juxtaposed against bold headlines. This was not a decision we took lightly or independently as researchers. In line with a reviewer’s helpful comments a retrospective decision was made to add some blurring to people in the background of photos. As Clark (2020) argues, a situated ethics of visual scholarship requires a commitment to involving participants in decision-making processes. We engaged in a deliberative discussion with the participant, and our discussions confirmed that family members featured had assented to their images being used in this and other situations. Where assent was not possible due to lack of capacity, we felt the decision to include the image was well thought out, ethically defensible, and not against the child’s best interests. Indeed, we would be more concerned by a decision to exclude them. As the artist and participant who created Figure 3 explained:

Ethically, I actually consider that it is important not to censor such images, in order to show the reality of the relationships that exist within families touched by rare genetic conditions, and to emphasize that the whole family is impacted by the issues raised and becomes part of a community of families sharing similar experiences.

As (Clark, 2020, p. 685) notes “visual researchers have been at the forefront of more critical approaches to normative assumptions around anonymity” leading to the emergence of an “ethics of recognition” as a relevant standpoint. That is, there is a need to be “aware of the desires of research participants themselves to not be hidden behind a cloak of anonymity” (Clark, 2020, p. 685). Disability Studies scholars have long highlighted how people with disabilities are often missing in visual representations, and images that visually reimagine disability are increasingly necessary (Garland-Thomson, 2005).

This speaks to one of the major challenges and limitations we encountered with collage as a method, in that collage-making is ultimately limited and shaped by the materials available and participants can struggle to “find” the images that are crucial to telling their stories, or be forced to adapt to what visual imagery is available from “ready-made” worlds (Raffaelli & Hartzell, 2016). The lack of imagery of disabled people in the public realm and mass media certainly challenged our participants and curtailed their ability to tell certain stories – as well as leading to the reliance on personal photos, which brought new ethical challenges as discussed above. There are obviously possibilities to collate more specific resource bundles on behalf of participants, but such curation requires collage-researchers to be reflexive about how that might inform what is produced, and we preferred to leave that curatorial agency to our participants.

Ultimately, what we take away from this process is not just the powerful artworks produced, but also the methodological atmosphere created. A strong sense of community emerged through the research, with the weekly meetings becoming a popular and highly sociable space with participants sometimes going to significant trouble to attend. This is similar to the recent conclusions drawn by Bailey and Woodall-Greene (2022, p. 1970) who suggest collaging collectively can foster a sense of group belonging through providing a means of “representing and discussing shared feelings of being misunderstood, unsupported, and weary”. People came to discuss their collage projects, to enjoy each other’s company, share frustrations about health and social care services – along with solidarities and suggestions for navigating these hurdles. People joined in alongside, and often despite, challenges at home.

Our adoption of arts-based methods has allowed us to remain engaged with our participants for longer, and more frequently, than would have been attractive to them in a more conventional research setting. The casual, pressure-free, no skills-required approach of collage created a space where people felt comfortable to explore aspects of family life and rare disease that have been more resistant to discussion in more interview and focus-group based work, but also, in comparison to textual creative practices, as, one of our participants reflects themselves:

I did an hour of collaging, which was wonderful. And I remember trying to do that with writing. I love to write, I love to share what I’ve written, but there is some barrier for me doing it: I want to write about this, I want to write about that, but I never do. But with collaging, it’s quicker, it’s accessible, you can do it for 15 minutes, you can sit down with a magazine and do some ripping! I’m finding it much easier, and when we spoke last time, I got really inspired and that’s when I did the collage … it brought so many feelings up that I wanted to write about, but I haven’t, and I can’t. But collaging about that feels much easier and accessible and straightforward.

This highlights the valuable potential collage as a method of qualitative inquiry offers in creating space for modes of representation, and ultimately, different knowledge. This is not about saying that collaging provides some unique insight into parents’ experiences and views that would not be revealed through a mobilisation of more conventional methods, but rather appreciating the depth, nuance, and richness which collage brought to the fore. It is also about finding new ways of offering representational agency to participants and creating affective and emotive ways for stories which may have gone unnoticed to travel and resonate in unexpected and unplanned ways (Parr, 2021).

As Bell (2013, p. 233) reflects, using collage as a participatory method is not a straightforward endeavour: “great care and sensitivity need to be exercised, time spent beforehand ‘setting the scene’, defining the context and aims, making it accessible for all concerned, establishing empathy and a location of trust”. However, from our perspective, and clearly from the perspectives of our participants, doing this work has been tremendously worthwhile, providing new ways of thinking about, understanding, and sharing the complex multifaceted experiences of families affected by rare conditions. By the end of the project we were convinced that collage, as a practice, allows people’s participation in research to exceed singular or reductive framings; our participants were there as artists exploring rare disease *with* us, rather than as people with rare disease in their families to be interviewed *by* us.

## Data Availability

Due to the highly personal, sensitive, and emotional nature of the qualitative data generated, and in order to respect participant’s preferences and consent, at this stage the rest of the collages, along with discussion group transcripts, are not being made publicly available beyond what has been published in this article. Interested parties are welcome to contact the corresponding author for further details.
